# Conventional and assisted suicide in Switzerland: Insights into a divergent development based on cancer‐associated self‐initiated deaths

**DOI:** 10.1002/cam4.6323

**Published:** 2023-08-09

**Authors:** Uwe Güth, Christoph Junker, Bernice Simone Elger, Constanze Elfgen, Giacomo Montagna, Andres R. Schneeberger

**Affiliations:** ^1^ Department of Breast Surgery Brust‐Zentrum Zürich Zurich Switzerland; ^2^ Faculty of Medicine University of Basel Basel Switzerland; ^3^ Federal Statistical Office Neuchâtel Switzerland; ^4^ Institute for Biomedical Ethics University of Basel Basel Switzerland; ^5^ Center for Legal Medicine University of Geneva Genève Switzerland; ^6^ Faculty of Medicine University of Witten/Herdecke Witten Germany; ^7^ Breast Service, Department of Surgery Memorial Sloan Kettering Cancer Center New York New York USA; ^8^ Department of Psychiatry University of California San Diego La Jolla California USA

**Keywords:** assisted dying, assisted suicide, cancer, end‐of‐life decision‐making, suicide

## Abstract

**Background:**

We tested the hypothesis of supporters of assisted dying that assisted suicide (AS) might be able to prevent cases of conventional suicide (CS).

**Methods:**

By using data from the Federal Statistical Office, we analyzed the long‐term development of 30,756 self‐initiated deaths in Switzerland over a 20‐year period (1999–2018; CS: *n* = 22,018, AS: *n* = 8738), focusing on people suffering from cancer who died from AS or CS.

**Results:**

While cancer was the most often listed principal disease for AS (*n* = 3580, 41.0% of AS cases), cancer was listed in only a small minority of CS cases (*n* = 832, 3.8% of CS cases). There was a significant increase in the absolute number of cancer‐associated AS cases: comparing four 5‐year periods, there was approximately a doubling of cases every 5 years (1999–2003: *n* = 228 vs.2004–2008: *n* = 474, +108% compared with the previous period; 2009–2013: *n* = 920, +94%; 2014–2018: *n* = 1958, +113%). The ratio of cancer‐associated AS in relationship with all cancer‐associated deaths increased over time to 2.3% in the last observation period (2014–2018). In parallel, the numbers of cancer‐associated CS showed a downward trend only at the beginning of the observation period (1999–2003, *n =* 240 vs. 2004–2008, *n =* 199, −17%). Thereafter, the number of cases remained stable in the subsequent 5‐year period (2009–2013, *n =* 187, −6%), and increased again toward the most recent period (2014–2018, *n =* 206, +10%).

**Conclusion:**

The assumption that, with the increasingly accessible option of AS for patients with cancer, CS suicide will become “superfluous” cannot be confirmed. There are strong reasons indicating that situations and circumstances of cancer‐associated CS are different from those for cancer‐associated AS.

## Graphical abstract

In order to test the hypothesis of supporters of assisted suicide (AS) that legalization of this procedure might be able to prevent cases of conventional suicide (CS), we tested the long‐term development of 30,756 self‐initiated deaths in Switzerland over a 20‐year period (1999–2018; CS: *n* = 22,018, AS: *n* = 8738), focusing on people suffering from cancer who died from AS or CS. While cancer was the most often listed principal disease for AS (*n* = 3580, 41.0% of AS cases), cancer was listed in only a small minority of CS cases (*n* = 832, 3.8% of CS cases). There was a significant increase in the absolute number of cancer‐associated AS cases in that there was approximately a doubling of cases every 5 years (1999–2003: *n* = 228, 2004–2008: *n* = 474, 2009–2013: *n* = 920, 2014–2018: *n* = 1958). In parallel, the numbers of cancer‐associated CS remained comparatively stable (1999–2003: *n =* 240, 2004–2008: *n =* 199, 2009–2013: *n =* 187, 2014–2018, *n =* 206). The assumption that, with the increasingly accessible option of AS for patients with cancer, CS suicide will become “superfluous” cannot be confirmed. There are strong reasons indicating that situations and circumstances of cancer‐associated CS are different from those for cancer‐associated AS.

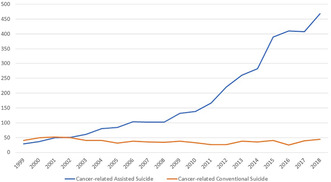

## 1 INTRODUCTION

The legalization of medical aid in dying, and in particular physician‐assisted suicide (AS), is one of the most debated topics in the field of medical ethics worldwide.^1^, ^2^, ^3^, ^4^, ^5^ In recent years, most Western countries have experienced a clear and steady increase in the acceptance of individual autonomy rights regarding the time of one's own death, particularly for patients suffering from a terminal illness and/or who experience unbearable and uncontrollable pain.^6^, ^7^, ^8^, ^9^, ^10^, ^11^ As part of this development, there are a growing number of countries which have altered or adjusted their laws to allow for different forms of assisted dying under certain conditions.^12^

Supporters of AS repeatedly bring up for discussion that AS might be able to prevent cases of conventional suicide (CS).^13^, ^14^, ^15^ Practical case vignettes are often presented to describe people who are suffering from an incurable somatic illness. In the late stages of the disease, some affected persons are plagued by the severity of their physical symptoms, and without hope for improvement of their situation, they only envision suicide as a viable next step. If at that point of the disease development they were prevented from accessing AS (i.e., in countries where it is not permitted), their only option to accomplish their wish, would be to pursue CS. Experts have pointed out that AS, carefully prepared in advance, accompanied by experienced helpers and possibly also in the presence of family and friends, represents a far more humane “solution” than the lonely and often more violent death (e.g., shooting, hanging, jumping from heights, and suicide by train) as seen with CS, which may also considerably traumatize both, family and friends as well as external bystanders who happen to witness the act of the suicide.

Following the arguments of supporters of assisted dying, access to AS should lead to a reduction in violent CS. Consequently, in Switzerland, where AS have increased considerably over the last 20 years,^16^ a reverse effect should therefore be observed for CS cases. However, to test the hypothesis that a reduction of CS is related to the widespread implementation of AS, only selected cases, which show comparable pre‐suicide conditions are suitable. Cases of suicide triggered by a late stage of cancer are most appropriate to conduct this examination.

Using data from the Federal Statistical Office (FSO), we present the long‐term development and trends of self‐initiated deaths (this term covers both, AS and CS) in Switzerland over a 20‐year period (1999–2018), focusing on people who suffered from cancer and died from AS or CS. To our knowledge, our time‐series analysis presents the longest period and highest number of cases ever reported on cancer‐associated AS and CS in medical literature.

## 2 PATIENTS AND METHODS

### 2.1 Data source

The Swiss cause of death statistics are derived from medical causes (principal disease, direct cause leading death, and comorbidities) reported by physicians who signed the death certificates. Diagnoses are recorded based on the International Classification of Diseases (ICD‐10) and are collected by the FSO according to the rules defined by the World Health Organization (WHO).^17^, ^18^ All aggregated data are treated anonymously and strictly confidentially and are subject to the provisions of the Swiss Federal Act on Data Protection (currently valid: SR 235.1).^19^ Publications on the cause of death statistics refer to persons who are legal residents of Switzerland, that is, on the permanent resident population regardless of nationality and place of death.

According to the currently valid ICD‐10‐based classification of the FSO, CS suicides are logged with a code from the sub‐chapter X60‐84 (“Intentional self‐harm”). AS cases are logged with code X61.8 as direct cause of death (as the ICD‐10 classification does not provide a separate code for AS, the FSO has given the established code X61 which is used for “Intentional self‐poisoning by and exposure to antiepileptic, sedative‐hypnotic, antiparkinsonism and psychotropic drugs, not elsewhere classified” its own supplementary digit: X61.9). In all AS cases, the illness or disease leading to the suffering was coded as the underlying cause of death.^18^ In this sense, assisted suicide is considered as being the last resort taken at the end of a serious disease.^18^ In addition, in all cases, physicians who signed the death certificate are requested to report the variables “principal disease” and “comorbidities.^17^ In unclear cases, the cancer subtypes associated with AS were matched by the FSO with data from the cantonal cancer registries.

For this study, we analyzed all death cases in Switzerland from 1999 to 2018; in this 20‐year period 1,261,923 people died (Table 1). Typical for an aging Western population, the median age at death was high and increasing (for men in 1999: 76 years, in 2018: 80 years; for women in 1999: 84 years, in 2018: 86 years). In the study period, 323,610 cancer‐related death cases were recorded. The proportion of deaths caused by cancer remained stable over time and was approximately 30% of the age‐standardized mortality rate^20^ (Table 1). People whose cause of death was cancer‐related were younger than those who died from a different non‐accidental cause (total population: 74 vs. 84 years; men: 74 vs. 81 years; women: 75 vs. 87 years).

**TABLE 1:** Conventional and assisted suicide in Switzerland (1999–2018) with particular consideration of cancer as cause of the self‐determined death.

Time period	Entire period 1999–2018	1999–2003	2004–2008	2009–2013	2014–2018
**All death cases** (men and women)	1,261,923	311,097	303,909	316,350	330,567
Median age at death	82	80	81	82	83
Cause of death: cancer (% on all deaths)	323,610 (25.6)	76,826 (24.7)	78,938 (26.0)	81,964 (25.9)	85,882 (26.0)
Median age at death	74	74	74	75	75
**Conventional suicide** (% on all deaths)	22,018 (1.8)	6155 (2.0)	5453 (1.8)	5250 (1.7)	5160 (1.6)
% change compared with the previous period			−11.4	−3.7	−1.7
Chi‐squared statistic (*p*‐value)			28.2 (*p* < 0.001)	16.6 (*p* < 0.001)	9.9 (*p* = 0.002)
Median age at death	52	51	51	53	55
Sex distribution: men (%)	72.5	72.1	71.2	73.2	73.4
Cancer reported as underlying disease (% on all conventional suicide cases)	832 (3.8)	240 (3.9)	199 (3.6)	187 (3.6)	206 (4.0)
% change compared with the previous period			−17.1	−6.0	+10.1
Chi‐squared statistic (*p*‐value)			not reported	not reported	not reported
Median age at death	72	73	72	73	72
Sex distribution: percentage of men (%)	79.1	75.8	81.9	77.0	82.5
**Assisted suicide** (% on all deaths)	8738 (0.7)	582 (0.2)	1161 (0.4)	2175 (0.7)	4820 (1.5)
% change compared with the previous period			+99.5	+87.3	+121.6
Chi‐squared statistic (*p*‐value)			206.7 (*p* < 0.001)	270.4 (*p* < 0.001)	897.4 (*p* < 0.001)
Median age at death	78	75	76	78	80
Sex distribution: percentage of men (%)	42.8	41.9	44.3	42.4	42.7
Cancer reported as underlying disease (% on all assisted suicide cases)	3580 (41.0)	228 (39.2)	474 (40.8)	920 (42.3)	1958 (40.6)
% change compared with the previous period			+107.9	+94.1	+112.8
Chi‐squared statistic (*p*‐value)			80.0 (*p* < 0.001)	127.6 (*p* < 0.001)	333.4 (*p* < 0.001)
Median age at death	73	71	71	74	75
Sex distribution: men (%)	50.9	48.7	49.2	52.6	50.8

### 2.2 The development of self‐initiated deaths in switzerland over time

#### 2.2.1 Conventional suicide

Between 1999 and 2018, a total of 22,018 cases of CS were registered among residents living in Switzerland. This was equal to 1.8% of all death cases (Table 1; ranges over the entire 5‐year observation subperiod: 1.6%–2.0%). From the first observation period (1999–2003) to the subsequent one (2004–2008), the number of cases fell considerably (−11.4%; chi‐squared statistic: 28.2; *p* > 0.001). Since 2010, the number of CS cases has been stable around 1030 cases per year (2010–2018, range 1005–1075). Males committed CS significantly more often than females, and these cases accounted for 72.5% of CS deaths. The median age at death was 52 years.

**FIGURE 1:**
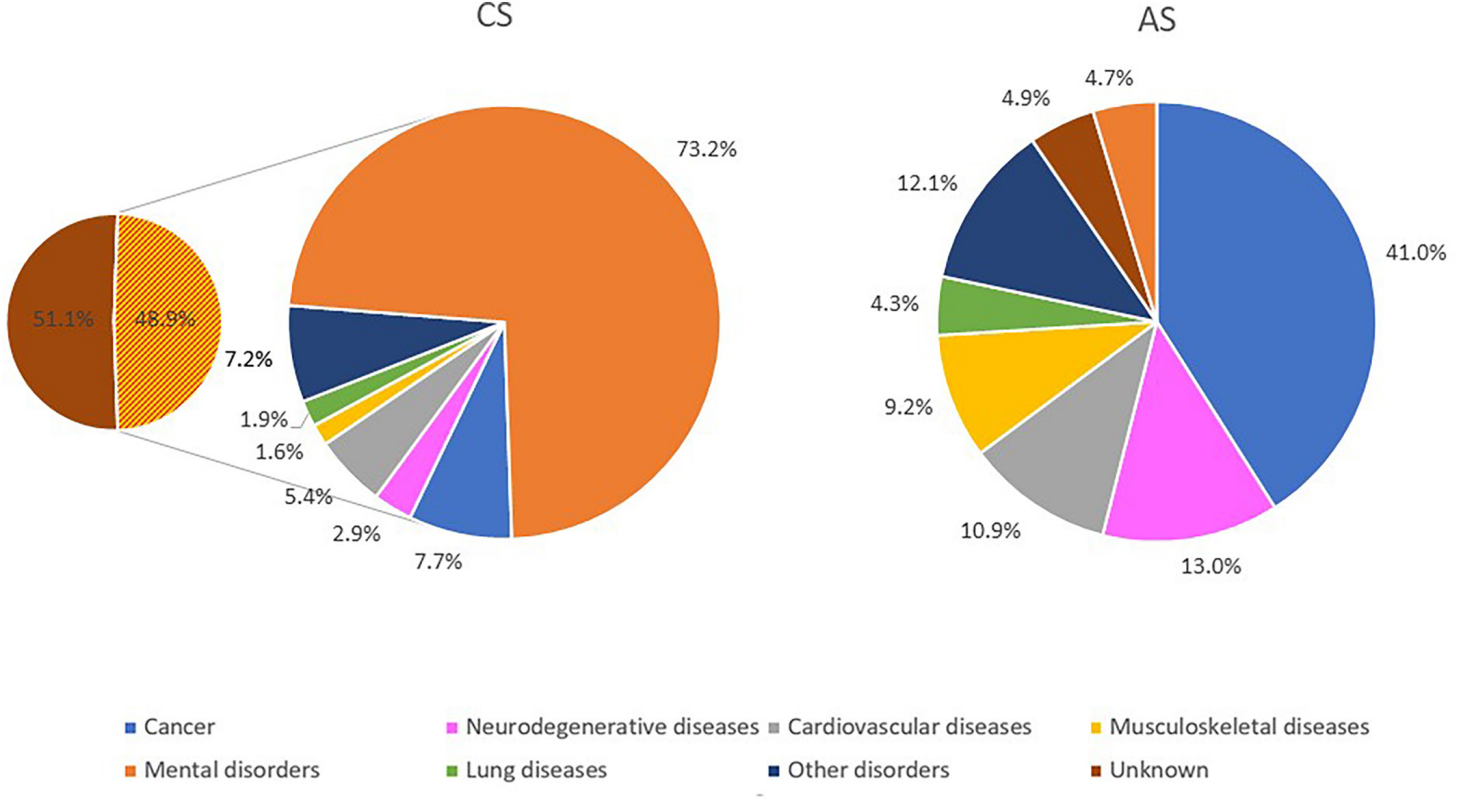
Conventional and assisted suicide cases in Switzerland 1999–2018: distribution of underlying conditions and/or diseases. Small left pie chart, section with orange and red stripes: In 48.9% of 22′018 conventional suicide cases, the FSO Swiss cause of death statistics included information about underlying conditions and/or diseases. These cases form the large left pie chart (“CS”).

CS reporting counts “suicide” as principal disease and the suicide method as direct cause of death. In 51.1% of CS cases, the reporting did not contain any information about comorbidities (Figure 1). If information was available, 73% of entries cited mental disorders. The most common CS method was hanging (29.0%): 31% of men and 23.7% of women used this method (Table 3). However, there were more striking gender‐specific differences in terms of two other most commonly used methods: while 30.5% of men committed CS by firearms, only 3.5% of the women used this form. In contrast, the most common CS form among women was poisoning (24.1%), followed by hanging, which was the second most frequent type.

#### 2.2.2 Assisted suicide

During our 20‐year observation period, the FSO registered a total of 8738 cases of AS. Over time, the number of AS rose significantly (Table 1), that is, for every 5‐year subperiod the number of AS cases somewhat doubled compared to the respective preceding 5‐year period.^16^ During the observation period, the percentage of AS among all death cases rose from 0.2% (1999–2003) to 1.5% (2014–2018). Generally older people chose AS (median age: 78 years), with a slight but stable predominance of women (56.9%; ranges over the four observation subperiods: 55.7%–58.1%).^16^

Upon further observation of the development of cases in recent years, we can see that the two forms of self‐initiated deaths have continued to converge in terms of quantity. In 2018, the number of annual AS reached its highest point during the years analyzed for this study at 1176 cases, making up 1.8% of all death cases. In that year, for the first time, cases of AS exceeded cases of CS (1176 AS cases vs. 1002 CS cases), and AS accounted for 54% of all self‐initiated deaths. In contrast, during the first year of our observation period in 1999, 63 cases of AS were reported compared with 1234 cases of CS, and AS accounted for just 5% of self‐initiated deaths.

### 2.3 Statistical analysis

Chi‐squared test was used to demonstrate the significant increase in AS cases over time, and to compare different suicide methods, both between men and women and between the two types of self‐initiated death. A *p*‐value <0.05 was considered significant.

## 3 RESULTS

### 3.1 The development of cancer‐associated self‐initiated deaths over time

#### 3.1.1 Assisted suicide

**FIGURE 2:**
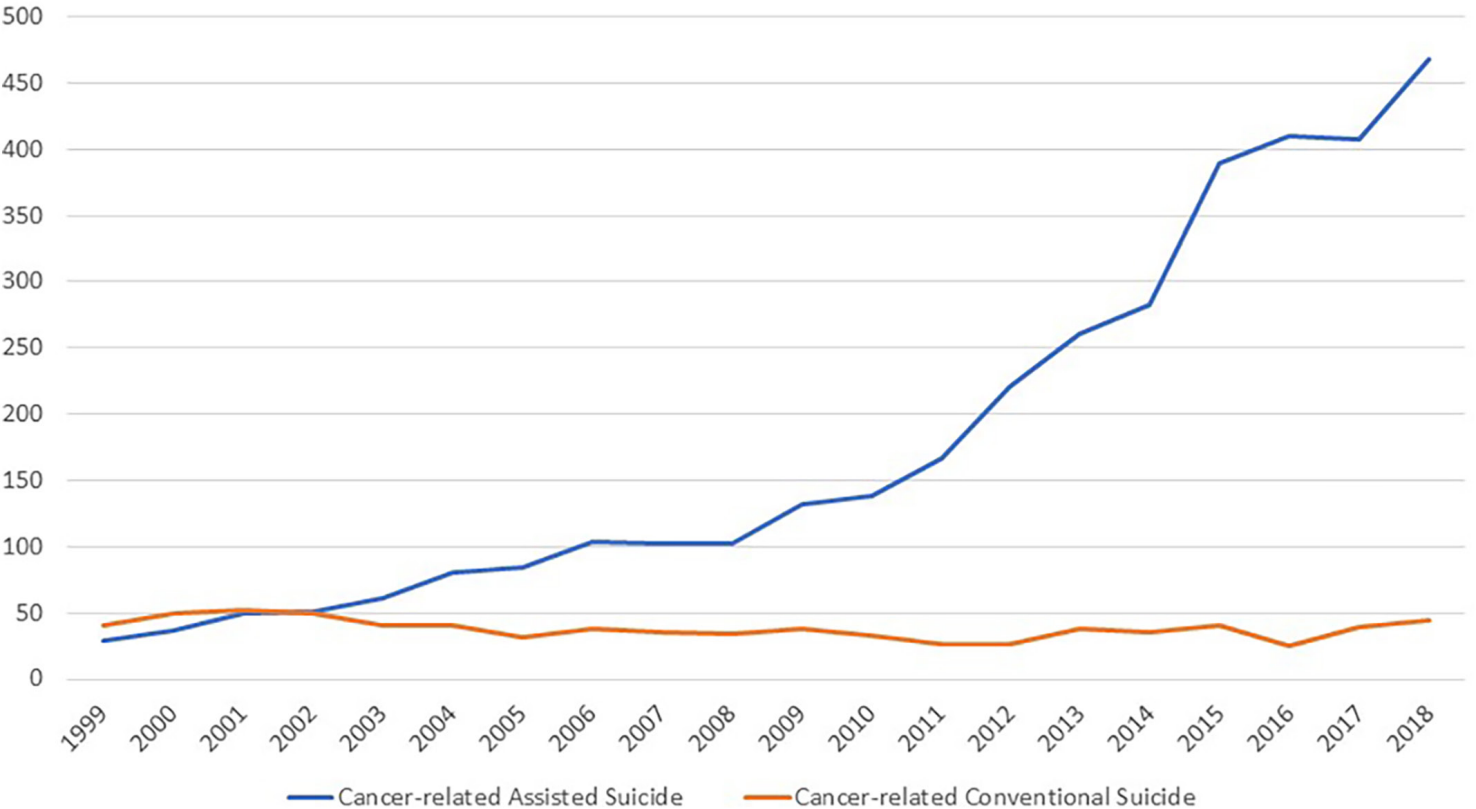
Development of the number of cancer‐associated conventional and assisted suicide cases in Switzerland 1999–2018.

Cancer was the most common reported principal disease for AS (*n* = 3580, 41.0%; Table 1). Moreover, the proportion of cases in which a malignant tumor was associated with AS remained relatively stable during each of the four defined time intervals (39.2%, 40.8%, 42.3%, and 40.6%). Parallel to the substantial rise of the total number of AS cases, there was also a significant increase in the absolute number of cancer‐associated AS cases: comparing the four 5‐year periods, there was approximately a doubling of cases every 5 years (1999–2003: *n* = 228; 2004–2008: *n* = 474, +108% compared with the previous period; 2009–2013: *n* = 920, + 94%; 2014–2018: *n* = 1958, +113%; Table 1, Figure 2).

The crude ratio of cancer‐associated AS in relationship with all cancer‐associated deaths increased over time for all cancer subtypes, ranging from 0.3% at the beginning of the study period (1999–2003) to 2.3% in the last (2014–2018).

Individuals who died from AS and had a malignant tumor listed as principal disease were considerably younger than those who died from AS with a reported other principal diagnosis (median ages at death: 74 years vs. 81 years). The sex ratio was nearly equal (men: 50.9% vs. women: 49.1%). Table 2 shows the distribution of AS by sex and for the four most common cancer subtypes (those with ≥350 AS cases): lung, breast, prostate, and colorectal cancer.

**TABLE 2:** Cancer‐associated assisted and conventional suicide in Switzerland (1999–2018) incl. data of the four most frequent types of cancer.

Time period	Entire period 1999–2018	1999–2003	2004–2008	2009–2013	2014–2018
**Men: All cancer death cases (% of all ♂ deaths)**	178,888 (29.3)	42,638 (28.3)	43,639 (29.7)	45,304 (29.7)	47,307 (29.6)
Median age at death	74	73	74	74	75
Lung cancer (% of all ♂ cancer death cases)	39,922 (22.3)	9931 (23.3)	10,055 (23.1)	9923 (21.9)	10,013 (21.2)
Prostate cancer (% of all ♂ cancer death cases)	26,527 (14.8)	6590 (15.4)	6479 (14.8)	6698 (14.8)	6760 (14.3)
Colorectal cancer (% of all ♂ cancer death cases)	17,961 (10.0)	4249 (10.0)	4391 (10.1)	4655 (10.3)	4666 (9.9)
**Male cancer‐associated assisted suicide (AS) cases**	1822 (1.0)	114 (0.3)	230 (0.5)	484 (1.1)	994 (2.1)
(% of all ♂ cancer‐associated deaths)					
% change compared with the previous period			+101.8	+110.4	+105.4
Chi‐squared statistic (*p*‐value)			36.6 (*p* < 0.001)	81.8 (*p* < 0.001)	157.2 (*p* < 0.001)
Median age at death	75	73	76	75	75
Prostate cancer (% of all ♂ AS cases)	367 (9.8)	22 (9.0)	56 (10.9)	93 (10.1)	196 (9.5)
Lung cancer (% of all ♂ AS cases)	239 (6.4)	25 (10.2)	24 (4.7)	55 (6.0)	135 (6.6)
Colorectal cancer (% of all ♂ AS cases)	200 (5.4)	11 (4.5)	19 (3.7)	58 (6.3)	112 (5.4)
**Male cancer‐associated conventional suicide (CS) cases** ^**b**^	659	182	163	144	170
Percentage of all ♂ CS cases	4.1%	4.1%	4.2%	3.8%	4.5%
Median age at death	74	74	74	75	73
Prostate cancer^a^	166	41	42	37	46
Lung cancer^a^	101	32	28	19	22
Colorectal cancer^a^	62	19	14	16	13
**Women: All cancer death cases (% of all ♀ deaths)**	144,722 (22.2)	34,188 (21.3)	35,299 (22.5)	36,660 (22.4)	38,575 (22.6)
Median age at death	75	75	75	75	76
Breast cancer (% of all ♂ cancer death cases)	27,233 (18.8)	6683 (19.5)	6705 (19.0)	6921 (18.9)	6924 (18.0)
Lung cancer (% of all ♂ cancer death cases)	20,438 (14.1)	3831 (11.2)	4689 (13.3)	5622 (15.3)	6296 (16.3)
Colorectal cancer (% of all ♂ cancer death cases)	15,018 (10.4)	3863 (11.3)	3670 (10.4)	3729 (10.2)	3756 (9.7)
**Female cancer‐associated assisted suicide (AS) cases** (% of all ♀ cancer‐associated deaths)	1758 (1.2)	117 (0.3)	235 (0.7)	436 (1.2)	970 (2.5)
% change compared with the previous period			+100.9	+85.5	+122.5
Chi‐square statistic (*p*‐value)			35.5 (*p* < 0.001)	53.4 (*p* < 0.001)	180.0 (*p* < 0.001)
Median age at death	72	67	71	73	73
Breast cancer (% of all ♂ AS cases)	385 (7.7)	29 (8.6)	51 (7.9)	100 (8.0)	205 (7.4)
Lung cancer (% of all ♂ AS cases)	269 (5.4)	9 (2.7)	36 (5.6)	76 (6.1)	148 (5.4)
Colorectal cancer (% of all ♂ AS cases)	187 (3.7)	11 (3.3)	30 (4.6)	41 (3.3)	105 (3.8)
**Female cancer‐associated conventional suicide (CS) cases** ^**b**^	173	58	36	43	36
% of all ♀ CS suicide cases	2.9%	3.4%	2.3%	3.1%	2.6%
Median age at death	64	67	59	65	63
Breast cancer^a^	55	18	17	9	11
Lung cancer^a^	11	2	6	1	2
Colorectal cancer^a^	13	4	3	4	2

Abbreviations: AS, assisted suicide; CS, conventional suicide.

^a^ The three most common cancer subtypes accounted for 50% in male cancer‐associated CS cases, and for 46% of female cancer‐associated CS.

^b^ We do not report the characteristics “% change compared with the previous period”, and “Chi‐square statistic (*p*‐value)” for male and female cancer‐associated conventional suicide. As the number of cases, especially among women, is too small for this, we entirely report the data in a descriptive fashion. A calculation of the chi‐squared values is not possible, because there is no reference variable for the conventional cancer‐associated suicide cases. In contrast, the reference variable for cancer‐associated assisted suicides is the total number of cancer deaths.

#### 3.1.2 Conventional suicide

Between 1999 and 2018, the FSO registered in total 832 CS cases in which a malignant disease had been listed as comorbidity. Compared with AS, cancer was thus reported much less frequently, that is, in only 3.8% of all CS cases (or 7.7% of cases in which an underlying disease was recorded). Among cancer‐associated CS cases, men were even more overrepresented (79%) than among non‐cancer‐associated CS cases (72.5%). Similarly to the overall group of all CS cases, the numbers of cancer‐associated CS showed a downward trend at the beginning of the observation period (1999–2003, *n =* 240 vs. 2004–2008, *n =* 199, −17%). Thereafter, the number of cases remained relatively stable in the subsequent 5‐year period (2009–2013, *n =* 187), but increased again toward the most recent period (2014–2018, *n =* 206, +10%). The median age of persons with cancer‐associated assisted suicide was 72 years. This was markedly older than the age of persons who committed non‐cancer CS (52 years), but similar to the age of all cancer‐associated AS (73 years) and comparable to that of all cancer‐associated deaths (74 years).

During the 20‐year observation period, shooting was the most frequent method to commit cancer‐associated CS (34.4% vs. 23.2% compared to non‐cancer‐associated cases, *p* < 0.001; Table 3). Hanging (20.5%) and poisoning (19.7%) were the next most frequent methods. While poisoning was still the most frequent suicide method in the first observation period (1999–2003, *n* = 68; 28.3% of cases), the number of cases approximately halved in the following 5‐year periods (*n* = 96, on average 32 cases per period), representing 16.2% of all cancer‐associated CS cases between 2004 and 2018.

**TABLE 3:** Conventional suicide methods in Switzerland (1999–2018). Comparison between the total group versus the cancer‐associated suicide cases.

Time period	Entire period 1999–2018	1999–2003	2004–2008	2009–2013	2014–2018
**All suicides**	22,018	6155	5.453	5250	5160
Hanging (%)	6405 (29.1)	1683 (27.3)	1530 (28.1)	1599 (30.4)	1593 (30.9)
Shooting (%)	5106 (23.2)	1700 (27.6)	1297 (23.8)	1117 (21.3)	992 (19.2)
Poisoning (%)	3080 (14.0)	934 (15.2)	749 (13.7)	743 (14.2)	654 (12.7)
Jumping from height (%)	2828 (12.8)	714 (11.6)	751 (13.8)	658 (12.5)	705 (13.6)
By train (%)	2215 (10.1)	481 (7.8)	498 (9.1)	578 (11.0)	658 (12.8)
Other methods (%)	2384 (10.8)	643 (10.5)	628 (11.5)	555 (10.6)	558 (10.8)
**All suicides, comparison by gender (♂/♀)**	15,981/6037	4439/1716	3907/1546	3845/1405	3790/1370
Hanging (% of ♂ suicides/% of ♀ suicides)	31.0/23.7	28.9/23.3	30.5/20.6	32.4/25.3	32.6/26.0
Shooting (% of ♂ suicides/% of ♀ suicides)	30.5/3.5	36.4/4.6	31.5/3.5	27.7/3.3	25.3/2.5
Poisoning (% of ♂ suicides/% of ♀ suicides)	10.5/24.1	11.0/26.0	11.0/24.5	10.6/23.8	9.4/21.8
Jumping from height (% of ♂ suicides/% of ♀ suicides)	10.5/18.8	9.2/17.8	10.8/20.4	10.2/18.8	12.0/18.3
By train (% of ♂ suicides/% of ♀ suicides)	8.9/13.0	6.7/10.7	7.5/12.8	10.1/13.4	11.6/15.8
Other methods (% of ♂ suicides/% of ♀ suicides)	8.6/16.9	7.8/17.6	8.7/18.2	8.9/15.5	9.1/15.7
**All cancer‐associated suicides** ^**a**^	832	240	199	187	206
Hanging (%)	171 (20.5)	55 (22.9)	47 (23.6)	29 (15.5)	40 (19.4)
Shooting (%)	286 (34.4)	66 (27.5)	75 (37.7)	69 (36.9)	76 (36.9)
Poisoning (%)	164 (19.7)	68 (28.3)	27 (13.6)	34 (18.2)	35 (17.0)
Jumping from height (%)	112 (13.5)	27 (11.3)	32 (16.1)	23 (12.3)	30 (14.6)
By train (%)	18 (2.2)	4 (1.7)	3 (1.5)	5 (2.7)	6 (2.9)
Other methods (%)	81 (9.7)	20 (8.3)	15 (7.5)	27 (14.4)	19 (9.2)

^a^ Due to the comparatively small number of female cancer‐associated suicide cases (1999–2003, *n* = 58, 2003–2008, *n* = 36, 2009–2013, *n* = 43; 2014–2018, *n* = 38), we have refrained to report the six suicide methods separately by gender.

## 4 DISCUSSION

The Swiss long‐term data on self‐initiated deaths shows that a relative reversal of suicide trends began to occur in 1999. While AS cases have increased significantly, CS cases at the time began to decrease. Since 2010, the number of CS has been stable around a value of about 1030 cases per year (2010–2018, range 1005–1075).

In order to be able to make a meaningful comparison between AS and CS, it is first necessary to clarify the general situations in which the respective forms of suicide take place. AS usually occurs in late stages of an incurable somatic disease that will lead to death in the foreseeable future. The most common risk factors for CS, on the other hand, is likely to be acute personal crisis situations or psychiatric illnesses.^21^, ^22^, ^23^ In many cases, these were not even recognized by the outside world as a situation requiring treatment before the act. This is also reflected by the fact that the Swiss FSO only registers comorbidities in about half of the CS cases; in 51% of the cases, the statistic forms did not contain any information about comorbidities, that is, either no illness was present, or no illness was known to the (in general a forensic) physician who had signed the death certificate. When an indication was present, 73% of them were mental disorders. However, differences between the suicide types exist not only in terms of underlying situations and associated diseases. The two forms also differ significantly by age and gender distributions. While AS in Switzerland tends to be chosen more often by women (57% of cases in the observation period 1999–2018), CS is committed primarily by men with significantly higher incidences, nearly 75% of all cases, than women (in this context, however, the gender paradox of suicidal behavior must also be noted: females have been found to have a disproportionately higher rate of suicide attempts than men).^24^, ^25^, ^26^ While AS is a predominantly geriatric phenomenon (the median age in the above‐mentioned 20‐year period was 78 years; if cancer‐associated AS cases are excluded, it was as high as 81 years), CS, on the other hand, is committed by significantly younger people (median age: 52 years).

It is precisely because both forms of self‐initiated deaths are each based on a variety of different situations and motivations, there is a general consensus among experts that the development of AS has taken place only to a very limited extent, if at all “at the expense” of CS, that is none or only a very slight shift of “classic” suicide cases to the AS group has taken place over time.^27^ While the increase in AS reflects a social development that increasingly recognizes it as an accepted option, the decrease in CS appears to be to a large extent attributable to increased prevention measures. These specifically attempt to prevent suicidal acts by providing assistance in personal crisis situations and mental disorders. In recent years, the willingness of people at risk to accept these offers has apparently increased.^28^, ^29^

### 4.1 Cancer and suicide

In order to properly study and quantify the association between cancer and suicide in larger cohorts, two approaches are of interest to discuss further. On closer inspection, however, both approaches show methodological weaknesses, which ultimately only allow a rough approximation of the phenomenon “cancer and suicide”.

Regarding the first approach, analyzing the number of suicides in a group of cancer patients,^30^, ^31^, ^32^, ^33^, ^34^, ^35^, ^36^ several epidemiological studies have examined the topic of “cancer and suicide” with analyses from large regional and national cancer registries.^30^, ^31^, ^32^, ^33^, ^34^, ^35^, ^36^ These studies have one essential weakness in common: information about the life situation in which suicide had been chosen was not provided. “Cancer patient” does not mean, after all, that the person actually suffered from this disease at the time of the suicide. There are also many people who have overcome the disease, who may be considered cured and for whom the cancer diagnosis made many years before plays no role in the decision to commit suicide. If these people commit suicide, this is not a “suicide within a cancer cohort”, but an “ordinary” suicide which can have a variety of reasons including non‐cancer‐related life crises or other severe other nonmalignant somatic and/or psychiatric diseases. In a previous study on the subject of “Suicide of Breast Cancer Patients”, the Basel study group led by the first author of this paper (U.G.), evaluated the cases of women who committed suicide by using the psychological autopsy method. This case‐specific and individual‐centered approach revealed the background and motivation of the suicide in each individual case. The study showed that in one third of the reported cases somatic and psychiatric comorbidities, which were existent many years before the cancer diagnosis, were clearly recognized as the triggering factor for suicide.^37^

There is a further weak point in this approach. As there are certain risk and lifestyle factors which not only predispose people toward a certain type of cancer, but may also predispose these same people toward suicide (e.g., in the case of lung cancer: being male, older age, addictive behavior involving heavy tobacco and/or alcohol consumption, and/or substance abuse), the cancer disease cannot be considered to represent an independent risk factor for suicide.^30^, ^31^, ^32^, ^33^

In summary, it must be stated that epidemiological studies using this approach may give an overview of large cohorts of patients but are unable to provide needed details about a target variable that is so strongly influenced by individual factors in the way that suicide is.

### 4.2 Cancer‐associated assisted and conventional suicide in Switzerland

In the present study, we analyzed the association between CS and cancer using a second approach: to analyze a group of individuals who have committed suicide, and then to further analyze those cases in this group in which cancer was the most probable trigger to suicide.^38^, ^39^

This method also has potential weaknesses. For example, the physicians who completed the death certificate may not have been aware of cancer as a possible cause of suicide. On the other hand, we can assume that cancer diseases many years in the past, for which the influence on the performed suicide were either not known then, or seemed very unlikely, were not listed in the death statistics form. Of course, this is different for late‐stage and terminal cancer patients. In these cases, the disease takes such an important part in a person's life that it is naturally known to both, the patient's family and friends as well as the doctor. In these cases, the probability is higher that the disease was also noted on the death certificate. Although it is not certain that the cancer was the main or central reason for CS, there is a high degree of plausibility. It is important to note here that critics of assisted dying have often emphasized that the main cause in many suicide cases was not the severe somatic illness, but a depressive circumstance and thus potentially treatable trigger for the desire for death and the suicidal act.^5^, ^40^, ^41^ The median age of the people in whom cancer was noted as comorbidity associated with CS on the death statistic form can also be taken as an indicator that patients who were especially in the late and final stages of their disease are actually represented in the cancer‐associated CS group reported in our study: the age of 72 years was significantly above the median age of the non‐cancer CS cases (this is 52 years) and close to those of cancer‐associated AS (73 years), as well as cancer‐related deaths in general (74 years).

Our study shows a marked increase in cancer‐associated AS over the 20‐year observation period (1999–2018). Cancer‐associated CS suicide, on the other hand, was a comparatively rare phenomenon: 3580 cases with cancer‐associated AS were compared with 832 cases with cancer‐associated CS. At the beginning of our observation period (1999–2003), a decrease in the number of cases was still found, but in the subsequent 15 years the numbers remained relatively stable. As discussed above, it is a complex task to capture the phenomenon of suicide, suicide rates, and the conditions and diseases underlying suicides in cause‐of‐death statistics. Both unrecognized and underreported suicide cases, as well as other cases in which the motivation underlying the suicide was unknown/unrecognized play a role.^42^, ^43^, ^44^ Comparisons in an international context are often only possible to a limited extent. Han et al. found a decreasing trend of cancer‐related CS in the USA during the same 20‐year observation period (1999–2018), and this was in contradiction to an increasing trend of overall CS rate.^45^ In this study, the rate in which cancer was identified as contributing cause of CS was 0.9%. In contrast, our Swiss data shows a rate of 3.8%. The different rates can be explained by two factors:

1. In general, there is a high degree of underreporting in suicides^43^; however, if different methods of recording suicides are used in different countries, the rate of underreporting may differ again in various national statistics.
2. In some cases of cancer‐related CS, not the malignant disease but an accompanying depressive circumstance was interpreted as decisive for the suicide and thus not cancer, but depression was coded as underlying disease for CS.

The facts revealed in our analysis moves us clearly in the direction of concluding that the two entities under study are only comparable to a limited extent. The expansion of AS is an indicator that its social acceptance has increased; over the past 25 years, it has become a valid and non‐stigmatic choice for many people. Consequently, this development is also reflected in a significant increase in the number of cases of cancer‐associated AS. The assumption that over the years, with the increasingly open option of AS for patients with cancer, CS suicide will become “superfluous” cannot be confirmed.^27^ This brings us back to the thesis of the proponents of AS: why should persons shoot themselves, resort to hanging, jump from great heights to imminent death, or throw themselves in front of a train, if the mutually planned, safe and well‐organized option of AS is available? We must conclude that the situation and motivations for cancer‐associated CS seem to be clearly different from those for cancer‐associated AS. Surprisingly, even the number of cancer‐associated CS cases in which the suicide had been committed by poisoning (which is the suicide method of AS) has not changed over the past 15 years; the percentage of people who committed CS by poisoning remained unchanged at approximately 16%.

We can only speculate about the reasons for the continuing stable case numbers of cancer‐associated CS:

1. Steck et al. have shown that AS in Switzerland is mainly chosen by educated people living in a well‐off urban environment.^46^ It could be that for people who do not essentially live in this social environment, AS is less familiar and less understood socially as a selectable option.
2. For AS, patients must become “active”. They must contact a right‐to‐die organization and/or a physician who supports the wish to die. This requires a minimum of mental and physical strength, which some patients in late stages of their disease may no longer be able to muster.
3. In some patients, there is not only a “neutral” desire for death. Cancer is associated with personal failure, and suicide then represents not only the end of life, but a deliberately violent act of physical destruction.^47^

## AUTHOR CONTRIBUTIONS

**Uwe Güth:** Conceptualization (lead); data curation (supporting); formal analysis (lead); investigation (lead); methodology (lead); project administration (lead); resources (lead); software (equal); supervision (lead); validation (lead); visualization (lead); writing – original draft (lead); writing – review and editing (lead). **Christoph Junker:** Conceptualization (equal); data curation (lead); formal analysis (equal); funding acquisition (equal); investigation (equal); methodology (equal); project administration (supporting); resources (supporting); software (lead); supervision (equal); validation (equal); visualization (supporting); writing – original draft (supporting); writing – review and editing (supporting). **Bernice Simone Elger:** Conceptualization (supporting); data curation (supporting); formal analysis (supporting); funding acquisition (supporting); investigation (supporting); methodology (supporting); project administration (supporting); resources (supporting); software (supporting); supervision (supporting); validation (supporting); visualization (supporting); writing – original draft (supporting); writing – review and editing (equal). **Constanze Elfgen:** Conceptualization (supporting); data curation (supporting); formal analysis (supporting); funding acquisition (supporting); investigation (supporting); methodology (supporting); project administration (supporting); resources (supporting); software (supporting); supervision (supporting); validation (supporting); visualization (equal); writing – original draft (supporting); writing – review and editing (equal). **Giacomo Montagna:** Conceptualization (supporting); data curation (supporting); formal analysis (supporting); funding acquisition (supporting); investigation (supporting); methodology (supporting); project administration (supporting); resources (supporting); software (supporting); supervision (supporting); validation (supporting); visualization (supporting); writing – original draft (supporting); writing – review and editing (supporting). **Andres R. Schneeberger:** Conceptualization (equal); data curation (supporting); formal analysis (supporting); funding acquisition (supporting); investigation (supporting); methodology (equal); project administration (supporting); resources (supporting); software (supporting); supervision (supporting); validation (supporting); visualization (equal); writing – original draft (equal); writing – review and editing (equal).

## FUNDING INFORMATION

There was no financial support for our research.

## CONFLICT OF INTEREST STATEMENT

The authors have declared no conflicts of interest.

## Data Availability

We present data of the Swiss Cause of Death Statistics which are collected by the Federal Statistical Office. All aggregated data are treated anonymously and strictly confidentially and are subject to the provisions of the Swiss Federal Act on Data Protection.

## References

[1] Borasio GD , Jox RJ , Gamondi C . Regulation of assisted suicide limits the number of assisted deaths. Lancet. 2019;393(10175):982‐983. 10.1016/S0140-6736(18)32554-6 30797600

[2] Lo B . Beyond legalization ‐ dilemmas physicians confront regarding aid in dying. N Engl J Med. 2018;378(22):2060‐2062. 29847753 10.1056/NEJMp1802218

[3] Gamondi C , Borasio GD , Limoni C , Preston N , Payne S . Legalisation of assisted suicide: a safeguard to euthanasia? Lancet. 2014;384(9938):127. 10.1016/S0140-6736(14)61154-5 25016988

[4] Snyder Sulmasy L , Mueller PS , Ethics, Professionalism and Human Rights Committee of the American College of Physicians . Ethics and the legalization of physician‐assisted suicide: an American College of Physicians Position Paper. Ann Intern Med. 2017;167(8):576‐578. 28975242 10.7326/M17-0938

[5] Sprung CL , Somerville MA , Radbruch L , et al. Physician‐assisted suicide and euthanasia: emerging issues from a global perspective. J Palliat Care. 2018;33(4):197‐203. 29852810 10.1177/0825859718777325

[6] European European Values Study . Justfiable: Euthanasia. Accessed November 8, 2022. https://www.atlasofeuropeanvalues.eu/maptool.html

[7] Cohen J , Marcoux I , Bilsen J , Deboosere P , van der Wal G , Deliens L . Trends in acceptance of euthanasia among the general public in 12 European countries (1981‐1999). Eur J Public Health. 2006;16(6):663‐669. 16641157 10.1093/eurpub/ckl042

[8] Cohen J , Van Landeghem P , Carpentier N , Deliens L . Public acceptance of euthanasia in Europe: a survey study in 47 countries. Int J Public Health. 2014;59(1):143‐156. 23558505 10.1007/s00038-013-0461-6

[9] Emanuel EJ , Onwuteaka‐Philipsen BD , Urwin JW , Cohen J . Attitudes and practices of euthanasia and physician‐assisted suicide in the United States, Canada, and Europe. JAMA. 2016;316(1):79‐90. 27380345 10.1001/jama.2016.8499

[10] O'Neill C , Feenan D , Hughes C , McAlister DA . Attitudes to physician and family assisted suicide: results from a study of public attitudes in Britain. J Med Ethics. 2002;28(1):52. 11834762 10.1136/jme.28.1.52 PMC1733504

[11] Brauer S , Bolliger C , Strub JD . Swiss physicians' attitudes to assisted suicide: a qualitative and quantitative empirical study. Swiss Med Wkly. 2015;145:w14142. 25999298 10.4414/smw.2015.14142

[12] Mroz S , Dierickx S , Deliens L , Cohen J , Chambaere K . Assisted dying around the world: a status quaestionis. Ann Palliat Med. 2021;10(3):3540‐3553. 32921084 10.21037/apm-20-637

[13] Exit. Deutsche Schweiz . What Does Physician Assisted Suicide Mean? Updated July 2022. Accessed November 8, 2022. https://exit.ch/en/englisch/faq/

[14] Dignitas. Submission 67 by Dignitas to Medical Services (Dying with Dignity) Exposure Draft Bill 2014. Parliament of the Commonwealth of Australia. August 20, 2014. Accessed November 8, 2022. https://www.aph.gov.au/DocumentStore.ashx?id=9d3ff5aa‐2732‐4402‐ba87‐3e7912088f1e&subId=299804

[15] Jones DA . Euthanasia, assisted suicide, and suicide rates in Europe. J Ethics Ment Health. 2022;11:1‐35.

[16] Montagna G , Junker C , Elfgen C , Schneeberger AR , Güth U . Long‐term development of assisted suicide in Switzerland: analysis of a 20‐year experience. Swiss Med Wkly. 2022;153:40010. 10.57187/smw.2023.40010 36971666

[17] Federal Statistical Office (FSO) . Cause of death statistics. Death and its main causes in Switzerland. Published 2018. March 30, 2021. Accessed November 8, 2022. https://www.bfs.admin.ch/bfs/en/home/statistics/health/state‐health/mortality‐causes‐death/specific.gnpdetail.2021‐0541.html

[18] Federal Statistical Office (FSO) . Cause of death statistics. Assisted Suicide and suicide in Switzerland. October 30, 2016. Accessed November 8, 2022. https://www.bfs.admin.ch/bfs/de/home/statistiken/kataloge‐datenbanken/publikationen.assetdetail.3902308.html

[19] Federal Assembly of the Swiss Confederation . Federal Act on Data Protection. Current version; in force: July 1, 1993; abrogation date: September 1, 2023. https://www.fedlex.admin.ch/eli/cc/1993/1945_1945_1945/en. Revised version; in force: September 1,2023. https://www.fedlex.admin.ch/eli/oc/2022/491/de

[20] Montagna G , Junker C , Elfgen C , Schneeberger AR , Güth U . Assisted suicide in patients with cancer. ESMO Open. 2022;7(1):100349. 35066409 10.1016/j.esmoop.2021.100349 PMC8789521

[21] Garcia de la Garza A , Blanco C , Olfson M , Wall MM . Identification of suicide attempt risk factors in a national US survey using machine learning. JAMA Psychiatry. 2021;78(4):398‐406. 33404590 10.1001/jamapsychiatry.2020.4165 PMC7788508

[22] McFarland DC , Walsh L , Napolitano S , Morita J , Jaiswal R . Suicide in patients with cancer: identifying the risk factors. Oncology (Williston Park). 2019;33(6):221‐226. 31219606

[23] Sinyor M , Tse R , Pirkis J . Global trends in suicide epidemiology. Curr Opin Psychiatry. 2017;30(1):1‐6. 27845946 10.1097/YCO.0000000000000296

[24] Cano‐Montalbán I . Sociodemographic variables most associated with suicidal behaviour and suicide methods in Europe and America. A systematic review. Eur J Psychol Appl Leg Context. 2018;10:15‐25.

[25] Freeman A , Mergl R , Kohls E , et al. A cross‐national study on gender differences in suicide intent. BMC Psychiatry. 2017;17(1):234. 28662694 10.1186/s12888-017-1398-8 PMC5492308

[26] Organization for Economic Cooperation and Development (OECD) . Suicides. In OECD Factbook 2014: Economic, Environmental and Social Statistics. 2014. Accessed November 11, 2022. doi:10.1787/factbook-2014-99-en

[27] Girma S . Is assisted suicide a substitute for unassisted suicide? Eur Econ Rev. 2022;145:104113.

[28] Wasserman D , Iosue M , Wuestefeld A , Carli V . Adaptation of evidence‐based suicide prevention strategies during and after the COVID‐19 pandemic. World Psychiatry. 2020;19(3):294‐306. 32931107 10.1002/wps.20801 PMC7491639

[29] Arensman E . Suicide prevention in an international context. Crisis. 2017;38(1):1‐6. 28256167 10.1027/0227-5910/a000461

[30] Zaorsky NG . Suicide among cancer patients. Nat Commun. 2019;14(10):207. 10.1038/s41467-018-08170-1 PMC6331593 30643135

[31] Robson A , Scrutton F , Wilkinson L , MacLeod F . The risk of suicide in cancer patients: a review of the literature. Psychooncology. 2010;19(12):1250‐1258. 20213857 10.1002/pon.1717

[32] Hem E , Loge JH , Haldorsen T , Ekeberg O . Suicide risk in cancer patients from 1960 to 1999. J Clin Oncol. 2004;22(20):4209‐4216. 15483032 10.1200/JCO.2004.02.052

[33] Misono S , Weiss NS , Fann JR , Redman M , Yueh B . Incidence of suicide in persons with cancer. J Clin Oncol. 2008;26(29):4731‐4738. 18695257 10.1200/JCO.2007.13.8941 PMC2653137

[34] Rahouma M . Lung cancer patients have the highest malignancy‐associated suicide rate in USA: a population‐based analysis. Ecancermedicalscience. 2018;12:859. 30174721 10.3332/ecancer.2018.859 PMC6113987

[35] Henson KE , Brock R , Charnock J , Wickramasinghe B , Will O , Pitman A . Risk of suicide after cancer diagnosis in England. JAMA Psychiatry. 2019;76(1):51‐60. 30476945 10.1001/jamapsychiatry.2018.3181 PMC6583458

[36] Kendal WS . Suicide and cancer: a gender‐comparative study. Ann Oncol. 2007;18(2):381‐387. 17053045 10.1093/annonc/mdl385

[37] Güth U , Myrick ME , Reisch T , Bosshard G , Schmid SM . Suicide in breast cancer patients: an individual‐centered approach provides insight beyond epidemiology. Acta Oncol. 2011;50(7):1037‐1044. 21861596 10.3109/0284186X.2011.602112

[38] Nie J , O'Neil A , Liao B , Lu C , Aune D , Wang Y . Risk factors for completed suicide in the general population: a prospective cohort study of 242, 952 people. J Affect Disord. 2021;282:707‐711. 33445097 10.1016/j.jad.2020.12.132

[39] Fang F , Fall K , Mittleman MA , et al. Suicide and cardiovascular death after a cancer diagnosis. N Engl J Med. 2012;366(14):1310‐1318. 22475594 10.1056/NEJMoa1110307

[40] Sulmasy DP , Finlay I , Fitzgerald F , Foley K , Payne R , Siegler M . Physician‐assisted suicide: why neutrality by organized medicine is neither neutral nor appropriate. J Gen Intern Med. 2018;33(8):1394‐1399. 29722005 10.1007/s11606-018-4424-8 PMC6082198

[41] Cheung G , Douwes G , Sundram F . Late‐life suicide in terminal cancer: a rational act or underdiagnosed depression? J Pain Symptom Manage. 2017;54(6):835‐842. 28807701 10.1016/j.jpainsymman.2017.05.004

[42] Tang S , Reily NM , Arena AF , et al. People who die by suicide without receiving mental health services: a systematic review. Front Public Health. 2021;9:736948. 35118036 10.3389/fpubh.2021.736948 PMC8804173

[43] Tollefsen IM , Hem E , Ekeberg O . The reliability of suicide statistics: a systematic review. BMC Psychiatry. 2012;12:9. 22333684 10.1186/1471-244X-12-9 PMC3350416

[44] Sheats KJ , Wilson RF , Lyons BH , Jack SPD , Betz CJ , Fowler KA . Surveillance for violent deaths ‐ National Violent Death Reporting System, 39 states, the District of Columbia, and Puerto Rico, 2018. MMWR Surveill Summ. 2022;71(3):1‐44. 10.15585/mmwr.ss7103a1 PMC8807052 35085227

[45] Han X , Hu X , Zhao J , Ma J , Jemal A , Yabroff KR . Trends of cancer‐related suicide in the United States: 1999‐2018. J Natl Cancer Inst. 2021;113(9):1258‐1262. 33464295 10.1093/jnci/djaa183

[46] Steck N , Junker C , Zwahlen M , Swiss NC . Increase in assisted suicide in Switzerland: did the socioeconomic predictors change? Results from the Swiss National Cohort. BMJ Open. 2018;8(4):e020992. 10.1136/bmjopen-2017-020992 PMC5905761 29666138

[47] Ludwig B , Dwivedi Y . The concept of violent suicide, its underlying trait and neurobiology: a critical perspective. Eur Neuropsychopharmacol. 2018;28(2):243‐251. 29254658 10.1016/j.euroneuro.2017.12.001 PMC5809305

